# Reliability and Validity of Observational Gait Analysis by Physical Therapists: Possibility of Verifying Accuracy and Improving Technology in Visual Measurement of Joint Angles

**DOI:** 10.1298/ptr.E10342

**Published:** 2025-07-12

**Authors:** Daiki MATSUZAKA, Koki WAGATSUMA, Takenori SHIMADA, Kenta IKUSHIMA, Hiroyuki FUJISAWA

**Affiliations:** 1 Department of Rehabilitation, IMS Meirikai Sendai General Hospital, Japan; 2 Graduate School of Health and Environment Sciences, Tohoku Bunka Gakuen University, Japan; 3 Faculty of Medical Science & Welfare, Tohoku Bunka Gakuen University, Japan

**Keywords:** Observational gait analysis, Reliability, Validity

## Abstract

Objectives: The reliability of observational gait analyses is generally considered poor or moderate. This is considered due to the drawbacks in the experimental designs and measurement methods used in related studies. This study examined the reliability and validity of physical therapists’ (PTs) visual joint angle estimation in gait analysis with a rigorous design. Methods: Twenty PTs were divided into 2 groups of 10 each based on their experience: <10 years and >10 years. They observed videos and still images of patients with gait disorders and visually estimated the joint angles at specific points during the gait cycle. Reliability was assessed using the intraclass correlation coefficient, and validity was assessed by a correlation analysis, which compared their estimates with reference values obtained from the digital video analysis. Results: Intra-rater reliability for visual estimation using video tended to exceed 0.6 for most indicators, whereas inter-rater reliability ranged from 0.04 to 0.59. For validity, correlation coefficients indicated an accuracy of 0.06–0.62 for video and 0.08–0.68 for still images. Conclusions: Although intra-rater reliability tended to be higher than inter-rater reliability, neither reached levels considered sufficiently reliable, and validity showed only limited agreement with reference values. These results highlight the limitations of visual estimation in joint angle assessment. Future efforts should focus not only on improving observational consistency but also on improving agreement with reference values through corrective training using still images.

## Introduction

Gait analysis is performed by physical therapists (PTs) to identify abnormalities in movement. The methods of gait analysis can be broadly divided into 2 types: instrumented gait analysis (IGA) and observational gait analysis (OGA). OGA involves the visual assessment and estimation of parameters such as step length and joint angles without utilizing equipment; this allows for immediate and convenient assessment of gait. Consequently, several PTs prefer OGA in clinical settings owing to time and environmental constraints^1,2)^. However, a previous study of PTs in daily clinical practice found that over 70% of PTs feel gait analyses are difficult, the main reason being observation accuracy^3)^.

Since OGA relies on human visual information and cognitive judgment, it is less accurate than IGA. OGA accuracy has been evaluated primarily in the context of the reliability and validity of visual estimation of joint angles during gait. In general, OGA reliability is low or moderate^4,5)^, and its validity is low^6)^, which limits its use in clinical settings. Moreover, studies on experience effects in gait analysis show mixed results; some found no effect, others favored less-experienced raters^4,7)^.

Ridao-Fernández et al^2)^. reported that the quality of the experimental designs used in studies thus far is questionable. They reviewed the quality of the experimental designs utilized in 18 studies using the COnsensus-based Standards for the selection of the Health Status Measurement INstruments (COSMIN) checklist. The COSMIN checklist rates experimental designs on four levels. Most were “poor,” with few rated “excellent” or “good,” raising validity concerns.

The first drawback in previous studies is the visual estimation method. Many studies used video recordings for reproducibility, allowing therapists to pause and play in slow motion^8–10)^. However, clinical gait assessments occur in real time, creating a gap between experimental conditions and practice. Slow-motion playback also makes it unclear whether accuracy reflects static or dynamic estimation.

The second drawback in the experimental design is the assessment scale used. Studies have investigated the reliability and validity of gait assessment scales^11,12)^. Nominal and ordinal scales have primarily been adopted to score these gait assessment scales. In the studies that used nominal scales, the format typically involved the selection of options such as “poor,” “normal,” and “excessive.” However, the criteria between the scales are ambiguous, and the reliability of the raters’ subjective judgments is a concern. In the studies that used ordinal scales, the angle criteria of each assessment item were established at each stage (the hip joint angle at initial contact: 21° or more extension: −2; 0 to 15° extension: −1; 0 to 10° flexion: 0; 6° to 45° flexion: 1; 46° or more flexion: 2). However, errors within this scale were not considered.

Despite OGA’s convenience, a gap may exist between reported and actual accuracy due to experimental design issues. This study examined OGA accuracy using the COSMIN checklist, evaluating static and dynamic estimation without pause/slow motion, measuring errors with continuous scales, and analyzing differences by experience.

## Methods

### Ethical considerations

This study was conducted in accordance with the tenets of the Declaration of Helsinki and approved by the Research Ethics Committee of Tohoku Bunka Gakuen University (Approval No. 22-19). The participants were given a written explanation of the purpose of the study and provided written informed consent. This research was supported by the 2023 research grant for physical therapy from the Japanese Society of Physical Therapy.

### Raters

Raters (PTs) were divided into 2 groups: <10 years (3.1 ± 2.1) and ≥10 years (17.2 ± 4.4), with 10 participants each. Eligible PTs worked in hospitals or clinics and confirmed their understanding via a questionnaire.

### Filming of the participants

Ten patients who were hospitalized for rehabilitation (4 with stroke and 6 with orthopedic disease) were the participants observed. The inclusion criteria for these patients were: ability to walk more than 5 m indoors with or without the use of walking aids or assistive devices, exhibited no dangerous physiological changes, such as blood pressure fluctuations, during exercise, and exhibited no problems with verbal comprehension.

### Gait video recording

The gaits of these patients were recorded using a digital video camera (Panasonic HC-VX992MS, Osaka, Japan; effective pixel count: approximately 8.29 million). The frame rate was set at 60 Hz. The camera was positioned 4.5 m from the walkway to capture the entire body of the patient, and its height was adjusted to the level of the patient’s greater trochanter. The length of the walkway was set at 12 m, based on previous motion analysis studies that used video cameras^13)^. Under these conditions, the patients entered the frame at the 3 m mark after starting to walk, walked 6 m, and then left the frame. For each patient, the gait speed was adjusted to a comfortable pace, and they were allowed to use assistive devices as needed. Gait parameters, such as the number of steps, time taken to walk 10 m, stride length, cadence, walking speed, and gait ratio, were measured.

Increased gait variability is a characteristic of gait disorders in patients with stroke and those with orthopedic disease^14,15)^. OGA reliability studies suggest gait cycle variability affects reliability, so this study standardized it for visual estimation^9,16)^. Specifically, gait was filmed from the front projection of the video camera, and the captured gait cycle was used as the target for visual estimation. Additionally, the video was edited to include one cycle before and after the target gait cycle; thus, each patient’s video contained three gait cycles. The target gait cycle for visual estimation was clearly indicated in the video.

### Assessment form

**Fig. 1.**
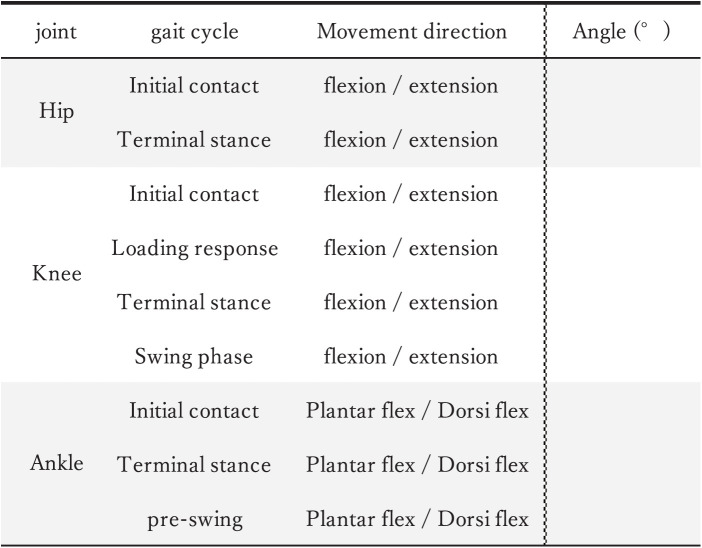
The assessment form provided to the raters

The assessment form was developed based on studies that investigated the kinematic variables of gait and included nine joint angles at critical events within the gait cycle^17–21)^ (Fig. 1). In the assessment form, the gait cycle was categorized using the Rancho Los Amigos classification system. The gait cycle was explained to the evaluators during the experiment and before the measurements to ensure uniform understanding and knowledge among participants.

### Procedure

The experimental design was structured according to the COSMIN checklist, adhering to the standards for verifying reliability and criterion-related validity^22)^. First, the raters watched an instructional video that provided an overview of the parameters to be measured and important considerations. The videos addressed the following points: 1) an overview of the experiment, 2) videos used in the experiment, 3) assessment parameters used in the experiment, and 4) instructions on how to respond to the assessment forms. The raters were instructed to check the direction of the joint angle (flexion or extension) for the relevant parameters on the assessment form and then assess the estimated joint angle (Fig. 1). They were also instructed to assess the angles at 5° intervals and not make any corrections. The raters were also instructed to record the projected angle as observed in the images. Moreover, 5° increments were used because, in clinical settings, PTs typically estimate joint angles in 5° units.

Subsequently, the raters were asked to randomly observe the gait videos of the 10 patients and record the measurements according to the assessment form format. The raters were asked to measure one index per gait video at the nine joint angles per gait video. They were asked to view the video 3 times for each joint angle. This yielded 27 measurements per video.

To verify the reliability, this assessment was performed on 2 separate days under the same conditions for the same rater. To eliminate the influence of the first assessment on the second, an interval is required between the days of measurement^23)^. In this study, the second assessment was performed at least 1 week after the first to ensure that the rater had sufficient time to forget the first assessment. Furthermore, to eliminate any effects of the video order, the order in which the videos were shown to the raters was randomized.

After completing the second set of measurements, the joint angles were visually estimated by the raters from the still images. Specifically, the joint angles were rated at specific points in the gait cycle using still images of the 10 patients. These still images correspond to the gait cycles rated during the video sessions over a 2-day period.

### Data and statistical analyses

The IGA results are used as reference values to investigate the accuracy of visual estimation using OGA. The recorded gait videos were loaded into a motion analysis application (Ugotoru version 4.4.13; Ugotoru, Tokyo, Japan). After identifying the timing of the target gait cycle, 9 joint angles formed by the attached markers were measured using ImageJ software (version 1.53, National Institutes of Health, Bethesda, MD, USA). All analyses were performed by a single designated individual. The method used had been validated in previous studies^24,25)^ compared with a three-dimensional motion analysis system. The digital video camera placement and analysis software procedures were strictly standardized. To ensure consistency in data analysis, all measurements were performed by a single rater to minimize bias.

Intra-rater reliability was assessed using intraclass correlation coefficients (ICC) (1,1) by verifying the consistency of each rater’s measurements between the first and second days. Inter-rater reliability was assessed using ICC (2,1) based on the mean joint angles reported by each evaluator on the first day.

Criterion-related validity was evaluated using Pearson’s product-moment correlation coefficient and Spearman’s rank correlation coefficient between the visual estimation from both videos and still images and the results obtained from the IGA. Each test was performed using R (ver. 4.2.1; R Foundation, Vienna, Austria), and statistical significance was set at p <0.05.

## Results

Table 1 shows the patient’s gait parameters in the gait video and the rater’s visual estimation of the angles of OGA. The patients were aged (mean ± standard deviation) 20.6 ± 1.7 years, with a mean height of 171.5 cm.

**Table 1.** The mean of the patient’s gait parameters and joint angle data and the rater’s visual estimation of the angles

Gait cycle parameters		Step length (m)	Number of steps (step)	Walking speed (m/s)		Walking speed (m/s)		Cadence (step/s)		Walking ratio
		0.4	14.4	0.52		0.52		1.4		0.0054
		(0.1)	(5.6)	(−0.02)		(−0.02)		(−0.52)		(0.0032)
		Hip		Knee				Ankle		
		Initial contact	Terminal stance	Initial contact	Loading response	Terminal stance	Swing phase	Initial contact	Terminal stance	Pre-swing
Joint angle ( °)	IGA	21.8	5.6	10.7	16.9	14.6	47.4	2.0	12.5	7.7
		(5.6)	(10.5)	(6.0)	(8.2)	(7.0)	(16.1)	(11.6)	(7.1)	(7.7)
	Less than 10 years group: visual estimation	26.5	−1.2	12.1	12.9	−9.3	32.8	0.1	3.9	2.6
		(4.2)	(9.0)	(4.6)	(6.0)	(5.2)	(10.8)	(5.3)	(7.7)	(2.5)
	More than 10 years group: visual estimation	9.9	9.7	7.0	8.6	9.9	17.7	7.7	12.5	7.8
		(2.2)	(3.6)	(3.1)	(2.6)	(3.1)	(5.9)	(3.0)	(4.2)	(2.9)

Mean (standard deviation)

IGA, instrumented gait analysis

In this study, the mean values of visually estimated joint angles from video observation (Table 1) and the estimation errors relative to IGA reference values (Table 2) were presented from different analytical perspectives. Table 1 shows the average of joint angle estimates across all raters, reflecting the overall trend in visual estimation. In contrast, Table 2 presents the absolute errors between each rater’s estimate and the reference values, and then uses the average of these individual errors to examine validity. Therefore, even for the same joint, the values in Tables 1 and 2 may not necessarily match. Table 3 shows intra- and inter-rater reliabilities.

**Table 2.** Errors and correlations between visual measurements and IGAs for video and still images

		Visual estimation in still images − IGA					
		Less than 10 years group			More than 10 years group		
		Errors (°)	Correlation coefficient	95% CI	Errors (°)	Correlation coefficient	95% CI
Hip	Initial contact	11 ± 10	0.06	−0.14−0.25	9 ± 6	0.15	−0.05−0.34
	Terminal stance	11 ± 8	0.35***	0.17–0.52	9 ± 6	0.34***	0.15−0.50
Knee	Initial contact	7 ± 5	0.45***	0.28−0.60	6 ± 5	0.62***	0.48−0.72
	Loading response	7 ± 6	0.55***	0.40−0.68	7 ± 5	0.62***	0.49−0.73
	Terminal stance	9 ± 6	0.52***	0.36−0.65	8 ± 7	0.52***	0.36–0.65
	Swing phase	20 ± 14	0.49***	0.33−0.63	19 ± 10	0.60***	0.46−0.71
Ankle	Initial contact	8 ± 6	0.45***	0.27−0.59	8 ± 6	0.61***	0.47−0.72
	Terminal stance	11 ± 8	0.24*	0.04−0.42	12 ± 8	0.43***	0.26−0.58
	Pre-swing	8 ± 6	0.30***	0.11−0.47	8 ± 6	0.31***	0.12−0.48
		**Visual estimation in still images − IGA**					
		**Less than 10 years group**			**More than 10 years group**		
		**Errors (°)**	**Correlation coefficient**	**95% CI**	**Errors (°)**	**Correlation coefficient**	**95% CI**
Hip	Initial contact	10 ± 7	0.08	−0.11−0.28	8 ± 6	0.23*	0.04−0.41
	Terminal stance	9 ± 9	0.33***	0.15−0.50	8 ± 6	0.52***	0.11−0.47
Knee	Initial contact	6 ± 5	0.55***	0.40−0.67	5 ± 4	0.68***	0.56−0.78
	Loading response	8 ± 6	0.62***	0.48−0.72	6 ± 5	0.64***	0.50−0.74
	Terminal stance	10 ± 11	0.54***	0.38−0.66	6 ± 5	0.68***	0.56−0.78
	Swing phase	12 ± 9	0.62***	0.49−0.73	13 ± 10	0.62***	0.49−0.73
Ankle	Initial contact	8 ± 6	0.50***	0.33−0.63	7 ± 6	0.52***	0.36−0.65
	Terminal stance	8 ± 7	0.49***	0.32−0.62	8 ± 7	0.45***	0.27−0.59
	Pre-swing	6 ± 4	0.52***	0.36−0.65	6 ± 5	0.49***	0.32−0.62

IGA, instrumented gait analysis; 95%CI, 95% confidence interval; *, p <0.05; ***, p <0.001

**Table 3.** Intra-rater and inter-rater reliability

		Less than 10 years group				More than 10 years group			
		ICC(1,1)		ICC(2,1)		ICC(1,1)		ICC(2,1)	
Hip	Initial contact	0.51	(0.35–0.64)	0.04	(−0.01–0.20)	0.65	(0.52–0.75)	0.06	(0.00–0.24)
	Terminal stance	0.72	(0.60–0.80)	0.48	(0.26–0.77)	0.85	(0.79–0.90)	0.59	(0.36–0.84)
Knee	Initial contact	0.74	(0.64–0.82)	0.21	(0.07–0.51)	0.77	(0.68–0.84)	0.38	(0.17–0.70)
	Loading response	0.58	(0.44–0.70)	0.29	(0.12–0.61)	0.70	(0.58–0.78)	0.43	(0.21–0.74)
	Terminal stance	0.50	(0.34–0.64)	0.22	(0.08–0.53)	0.75	(0.64–0.82)	0.21	(0.07–0.52)
	Swing phase	0.64	(0.51–0.75)	0.22	(0.08–0.53)	0.77	(0.68–0.84)	0.28	(0.10–0.61)
Ankle	Initial contact	0.54	(0.39–0.67)	0.36	(0.17–0.68)	0.75	(0.65–0.83)	0.47	(0.24–0.76)
	Terminal stance	0.46	(0.29–0.60)	0.09	(0.00–0.34)	0.59	(0.44–0.70)	0.33	(0.14–0.65)
	Pre-swing	0.43	(0.26–0.58)	0.13	(0.02–0.40)	0.65	(0.51–0.75)	0.18	(0.06–0.47)

Mean (standard deviation)

ICC, intraclass correlation coefficients

## Discussion

### Reliability

This study used ICC, a widely used metric for assessing consistency within ICC (1,1) and between raters ICC (2,1), to evaluate OGA accuracy.

Focusing first on intra-rater reliability, the >10-year group showed relatively higher ICC values compared with those reported in previous studies of OGA accuracy^7,9,16,26)^. This may be due to variations in visual estimation methods. Earlier studies allowed raters to assess joints freely, possibly leading to simultaneous multi-joint estimation. In contrast, the current study required the raters to estimate one specific joint angle per trial. This focused approach may have contributed to improved reliability by reducing the cognitive load and increasing attention to detail. Moreover, McGinley et al^4)^. suggested that reducing the number of rating items can reduce the cognitive load and improve reliability. This suggests that considering the cognitive load on raters when performing OGA is important. Therefore, improving rater focus may improve reproducibility. Furthermore, the >10-year group showed relatively better results than the <10-year group, likely because experienced raters are more skilled at recognizing joint angles due to their extensive clinical observation experience. Their exposure to a wide variety of patients may also have contributed to the development of internal criteria for identifying gait patterns and abnormalities. Meanwhile, both groups demonstrated low inter-rater reliability, which may be attributed to variability in visual processing ability, joint angle recognition, and estimation criteria shaped by differences in clinical experience and background. Specifically, in joint angle estimation tasks such as those used in this study, differences in how raters process visual information and apply their own internal standards can lead to inconsistent results, even when viewing the same visual stimuli.

These reliability results suggest that with increasing experience, visual estimates become relatively stable within each rater, while discrepancies may still occur between different raters. This trend is consistent with previous studies reporting higher intra-rater reliability than inter-rater reliability^7,9,16,26)^. However, according to the criteria proposed by Koo and Li^27)^, an ICC of ≥0.75 is considered indicative of good reliability, and even within the >10-year group in this study, some indicators did not meet this threshold. Therefore, it should be noted that even within the same rater, some degree of error may still occur in clinical application. Furthermore, the results suggest that the conduct of OGA should consider the rater’s cognitive load and the internal standards used for angle estimation. However, this study did not directly measure cognitive load, which should be addressed in future research.

### Validity

Static and dynamic visual estimation accuracies were assessed using still images and videos. Both groups had similar static estimation accuracy, but dynamic estimation varied: the <10-year group showed low correlations, while the >10-year group had low-to-moderate correlations. However, even the >10-year group lacked clinical reliability. Terwee et al.^28)^. stated that criterion-related validity requires a correlation of 0.7 or higher, which was not met in this study. Moreover, the human visual system is shown to have difficulty accurately detecting events above 12 Hz^29)^, which may also be relevant. These findings suggest that there are inherent limitations to the accuracy of visual estimates, regardless of the experience of the rater.

In both groups, focusing on the ankle joint, a notable difference between video and still images was observed, with video showing a sharp decline in accuracy compared to still images. This suggests that raters may struggle to account for the complex movements of the ankle during dynamic motion. In particular, the rapid changes in joint angles and momentary movements captured in videos may be difficult for raters to accurately perceive. In contrast, still images allow for the assessment of specific moments, reducing cognitive load and improving ankle observation accuracy.

The low validity observed for both video- and still-based estimation may be due in part to insufficient training of raters to calibrate their observation criteria with reference values. Particularly in dynamic observation, increased cognitive load may make it more difficult to achieve consistency with reference values. For clinical application, a stepwise training approach may be beneficial starting with still image estimation and gradually progressing to video-based estimation, with repeated comparisons to reference values to progressively reduce error. However, this study did not investigate whether such training would lead to improved validity, so this suggestion remains hypothetical and a direction for future research. To improve the validity of visual estimation, further studies are needed to clarify the effectiveness of such training interventions.

This study evaluated visual gait observation accuracy and its potential impact on abnormal gait detection. While OGA’s clinical role extends beyond angle estimation to detecting abnormalities, improving visual estimation may enhance detection accuracy. Future research should explore its effects on detection rates and clinical decisions. Furthermore, this study aimed to evaluate OGA accuracy as performed by PTs in clinical settings rather than gait analysis using software or artificial intelligence (AI). While software-based analysis provides more precise data, clinical assessment still relies strongly on visual observation, emphasizing the need to improve its accuracy. The findings of this study may contribute to enhancing OGA accuracy in clinical practice.

This study has several limitations. First, raters were grouped based on clinical experience, with a threshold of 10 years, to examine the effect of experience on observation accuracy. This classification was based on the hypothesis that accumulating clinical experience improves accuracy. Ericsson et al.^30)^. have suggested that approximately 10 years of experience is required to acquire advanced skills, supporting the use of 10 years as a benchmark for differentiating expertise in observation accuracy. However, further subdivisions, such as within 3 or 5 years of licensure, may be necessary to analyze the effect of experience in more detail. Second, the study included 10 patients in each group and used gait data from 10 raters, resulting in a total of 100 data points per gait cycle to ensure a sufficient sample size. However, to examine variations among raters in greater detail, the sample size may still be insufficient, necessitating further validation in future research. Third, this study used video-based gait observation, where the entire gait cycle was visible, and raters could observe the video multiple times, which differs from clinical assessments. In actual clinical settings, we sometimes observe from a certain distance with caution, while at other times, we observe closely while managing risks and within limited opportunities. Therefore, this method does not fully reflect actual clinical assessments. Fourth, the study included patients with cerebrovascular and orthopedic disorders but did not analyze how the characteristics of each condition influenced reliability and validity. Additionally, although patients with different gait speeds were evaluated, the effect of gait speed on observation accuracy was not examined. Future research should analyze these factors in greater detail to obtain more generalizable findings.

## Conclusions

This study investigated the observational accuracy of PT raters for an OGA in the context of reliability and validity for the visual estimation of joint angles. One strength of this study is that it was the first to evaluate visual estimation accuracy using continuous scales. Furthermore, this was the first study to assess both static and dynamic visual estimation accuracies. Moreover, this study aimed to evaluate the accuracy of visual joint angle estimation, which has implications for reevaluating the methods used in clinical OGA. The results showed that intra-rater reliability was generally higher than inter-rater reliability in both groups; however, neither reached sufficient levels to be considered fully reliable, suggesting that a certain degree of error is inherent in visual joint angle estimation. While the >10-year group showed relatively better performance than the <10-year group, the influence of clinical experience must be interpreted with caution. Both groups exhibited limitations in inter-rater reliability and validity, with insufficient correlation between estimated and reference values for both still images and videos. This suggests inherent limitations in angle estimation through observation. Therefore, rather than relying solely on years of experience, improving OGA accuracy may require training focused on refining angle recognition using still images and reducing discrepancies with reference values. In this process, priority should be given to correcting angle recognition from still images and minimizing errors relative to reference values rather than merely enhancing evaluation consistency. However, this study did not examine the effectiveness of such training, and future research is needed to clarify its impact on improving validity.

## Acknowledgments

The author would like to thank Yasuhide Nakayama (The Jikei University School of Medicine), Takeshi Kobayashi (Tohoku Bunka Gakuen University), and Kenichi Murakami (Tohoku Bunka Gakuen University) for their comprehensive instruction on related studies.

## Funding

This work was supported by the Japanese Society of Physical Therapy (JSPT23-024).

## Conflicts of Interest

The authors declare no conflicts of interest.
